# Extracts from a Lecture upon Recent Observation on Medical Subjects in Europe; Being Introductory to His Course at the Buffalo Medical College

**Published:** 1866-12

**Authors:** James P. White


BUFFALO

### Medical and Surgical Journal.

VOL. VI. DECEMBER, 1866. No. 5.

### Original Communications.

ART. I.—*Extracts from a Lecture upon Recent Observation on Medical Subjects in Europe; being Introductory to his course at the Buffalo Medical College.* BY PROF. JAMES P. WHITE.

[The following lecture delivered by Prof. James P. White since his return from Europe, is published in compliance with the wishes of his colleagues and pupils and many of his professional friends. Having during the summer published letters from Prof. W. describing the condition of medical affairs in Spain and Italy, we omit, at his request, everything relating to these countries in the lecture.—ED.]

GENTLEMEN:—Nearly one year since, I bade adieu to my class, many of whom it gives me pleasure to behold again in their places, for the purpose of renovating a system exhausted by unintermitting professional labors, and also for the purpose of visiting the medical schools and hospitals of Europe. This task has, by the blessing of Divine Providence, been accomplished without accident to my companions or myself, or the occurrence of any untoward circumstance to mar the pleasure of the trip. My most sanguine anticipations have been more than realized, and throwing aside the responsibilities of the practitioner, I have lived again the life of a student. During this time it has been my good fortune to visit a large number of the medical colleges and to see and hear lecture a great many medical men, most distinguished for their learning and ability. Throughout this extended journey, including Great Brittain and most of the countries in continental Europe, I have been treated with great hospitality, been welcomed with a degree of warmth and cordiality far exceeding my individual merits, and which was extended by brethren of a profession whose sphere of usefulness knows no political or geographical limits, but recognizes in the trans-Atlantic physician a co-worker in the great cause of ameliorating the condition of suffering humanity. And, gentlemen, much as I have enjoyed this tour in the old world, and much as I hope I have profited also in a professional point of view, by observations made in the various institutions which I have visited, I am delighted still more by your hearty welcome upon my return, and am most happy again to meet you and resume with renewed physical vigor, and to the best of my ability the duties here assigned me. Indeed, I can truly assert that the pleasure which I have derived from foreign travel culminated on each occasion in the hearty welcome extended to me by my friends, pupils and patients upon my return to the home of my boyhood and the theatre of professional labors of more than one-third of a century's duration.

The request has been repeatedly made, that, instead of a more formal or elaborate introductory discourse, this, our first interview, be occupied with a succinct account of the various objects which I have seen with most interest. In compliance with this desire I will hastily advert to some of the things which most attracted my attention, and note in passing some of the changes which have occurred since my previous visit. I may commence by saying that, leaving you the beginning of the present year, after a remarkably dangerous and tempestuous passage across the Atlantic, we were safely landed in Cherbourgh toward the last of January. Suffering greatly during this entire voyage, as well as on previous voyages, from sea-sickness, I regret to say that I was unable to turn my experience to any good account, and must confess that, after much inquiry and reflection, I now know of no remedy for that most vexatious affection, except to remain on shore. Perhaps I may add, in view of the department which it is my province to teach, that, the accoucheur who has been the victim of ten days' sea-sickness will be most certain to have a realizing appreciation of the discomforts of "morning sickness," and will thereafter be sure to lend a patient ear to the poor woman who claims his sympathy under such circumstances.

Proceeding to Paris by the way of Caen, I remained at the latter place a few days to see the various buildings and objects of interest in that venerable city. Fifteen years since I visited Rouen, also, to see M. Pouchet, the celebrated naturalist and physiologist. M. Pouchet, whom I found actively engaged in his favorite investigations, is slight in form, rather below the medium height, high forehead, with a piercing black eye, fine countenance, and about forty-five years old. It is to this physiological observer more than to all others, in my opinion, to whom the profession is indebted for experiments which establish the existence of spontaneous ovulation in all the mammalia. You are all aware, that but a few years since it was the accepted doctrine, that the influence of the male was necessary for the formation of the germ or ovule. That after a fruitful connection the ovary was excited to action, that then, and not till then, an ovule or germ was secreted or formed, which was subsequently transmitted to the uterus where it was matured and prepared for external life. Now this theory, with its kindred ones of menstruation and the formation of the corpora lutea, etc., etc., have been completely overturned by the experiments of MM. Bischoff, Rociborski, and especially by the labors of M. Pouchet of Rouen. M. P. gave me an early edition of his work upon "Spontaneous Ovulation," which, in my judgment, places the priority of his claim beyond all question, although its discovery has been claimed by others.

Leaving the city of Caen, in which William the Conquerer lived, and where his bones are still preserved, we proceeded by rail to the great French metropolis. Paris contains much which claims the attention of the medical observer as well as the lover of art and the votary of pleasure. In the study of anatomy, special, pathological and microscopic; in the perfection of diagnosis, especially in physical explorations; in experimental physiology; in many departments of surgery; in the diagnosis and treatment of the diseases pertaining to the female genital apparatus; in the opportunities afforded for investigating cutaneous diseases; and in many other dapartments which might be enumerated Paris offers unparalleled advantages. To the obstetrical student this city affords a larger number of labors which are made available as a means of instruction, than any other except Vienna. Here, also, by paying a small fee, the attendance of females in all the stages of pregnancy may be secured, who are willing to submit to an examination for all the tests or signs of that condition, thus rendering those who choose to profit by these opportunities, expert in detecting the various changes which the neck and orifice of the uterus undergo; the condition of the abdomen and mammæ, and more than all, in recognizing the intra-uterine sounds at an early date. In the great school of medicine, or "*ecole de medicine*," theoretical instruction is given during nearly ten months in the year, by twenty-four professors. The one-half lecture during the winter months and the remaining half during the summer *seicle*, as it ls termed. There are ordinarily three lectures, of one hour each, delivèred daily; commencing at ten or eleven, or so late as not to interfere with the clinics of the several hospitals, which ordinarily occupy from seven to nine in the morning. The faculty of this great school of medicine are appointed and paid by government, and until quite recently, after thoroughly sifting the qualifications of all applicants by the system of concouring. It may be remarked that the tickets, not only here, but at all the hospitals, also, are gratuitous. The faculty of this school alone can confer the degree of Doctor in Medicine, without which no one can practice his profession in the French Empire, without incurring the severest penalties of the law. In this faculty are always to be found some of the ablest men in France. The more distinguished of these professors ordinarily lecture to crowded rooms, there being not unfrequently from one to two thousand in the amphitheatre; whilst at other times, during the dull delivery of an uninteresting lecture, I have counted them when they did not exceed forty or fifty. It may not be uninteresting also to add, that, the accommodations for *sleeping* are very indifferent, as the seats are oak, uncushioned, and without backs; so that those only attend who expect to listen. Trosseau, Moreau, De Paul, Velpeau, Andral, Jobert, and some others, never lecture but with crowded benches. Most of the professors occupy a large, high chair, and deliver their lectures sitting; and the same remark holds true of the clinical teachers in the hospitals. There are, however, some exceptions. M. Orfila always lectures standing, gesticulates very much, and until recently maintained the high reputation as a chemist and teacher, which he had borne for almost half a century. M. Coste, the great physiologist, is a very animated lecturer and always stands in the delivery of his interesting lectures in the College de France. Trouseau, the author of a very valuable work upon Therapeutics, and who has also made several other contributions to science through the different journals, was appointed to his chair at an earlier age than any other man has ever been, and is even at the present time esteemed the most eloquent medical teacher in Paris. He is tall, with a large head, broad mouth and enunciates so distinctly that it has always seemed to me any one could understand him even though he might know nothing of the French language. During the month of August last, I had a long consultation with this celebrated practitioner and teacher, in the case of a friend and patient suffering from epilepsy, to which and kindred diseases Trouseau has devoted especial attention. He is vigorous and active, and I am happy to add, that although now a little more than sixty-five years old, is still thoroughly a man of progress. He resigned his chair in the faculty of the school of medicine only a few weeks since, under a resolution formed and adopted by his eloquent colleague, Orfila, and himself, when in middle life, predetermining *voluntarily* to retire at that age, without waiting until the public should cease to follow them, adding as he handed in his resignation, that "the labor of practice is already oppressive for more than sexigenarian shoulders; that of teaching is impossible." Would that all were equally conscious of the infirmities incident to advancing years. Malgaigne was an instance which may sometimes be met with of a man who writes and speaks well, without making a judicious practitioner in the same department in which he eloquently discourses. He was one of the surgeons to Hospital St. Louis, it is true, but in the two or three visits which I made in his ward, when at his zenith, I found scarcely a dozen following him, although he had a fine field for displaying practical talent, did he possess it.

It is not necessary for the student to attend any of the lectures in the ecole de medicine in order to entitle him to an examination before its faculty. This is especially fortunate, for the chairs being held for life, some of them are now, and probably always will be, occupied by superanuated old men who are utterly unfit for their positions, and yet unwilling to relinquish its emoluments. To obviate this defect, private courses are given by enterprising, ambitious men upon all the specialties, thus enabling the student to supply, for a small sum, any imperfections which there may be in the instructions at the college. Thus we find such men as Chailly, and Cazzeau, and De Paul, and Campbell, and Pagot and others, all of whom are distinguished authors of obstetrical works, lecturing for many years for a nominal compensation to private classes, as the best means of keeping themselves posted, and actively alive to everything new in the profession, and ready whenever a vacancy may occur in any of the chairs, to enter the list of concourants. The means of theoretical teaching are rendered greatly more perfect in consequence of the extensive museum attached to the ecole de medicine and the *muse dupuytren* which is in the vicinity of the school of medicine. But it is not theoretical teaching which has made Paris so celebrated as a school of medicine. To the great proportion of those pursuing their studies here, especially that respectable and numerous class of medical men who have already taken their degrees in some other country, the clinical, bed-side, or hospital teachings and observations are much the most attractive. The large hospitals of Paris afford ample material for illustrating every variety of disease. Permit me to read the following extracts from a letter written fifteen years since when I was upon the spot, and carefully collected the statistics it contains:

"The number of hospitals and hospices in Paris is very large, receiving annually nearly 80,000 patients. The number of indigent who every year receive gratuitous prescriptions and other assistance without becoming the inmates of any of these charitable institutions is also large. These institutions are divided into two classes. First, special hospitals, appropriated to the reception and treatment of particular classes of diseases; as for example, cutaneous affections, diseases of children, insanity, etc. Secondly, general hospitals, to which are sent all surgical and medical cases for which there is not a special hospital provided. Again, the large wards of the latter are so classified that not only are separate apartments assigned to the different sexes, but each "salle" or ward is in many instances destined for the reception of some specialty, as diseases of the lungs, heart, etc., as the physician or surgeon having charge of it may be considered most expert in the diagnosis and treatment of that disease with which the patient is afflicted. This distribution is easily effected through the agency of the "*Bureau Central*," which has a general supervision over all the hospitals, and to which most of the applications for admission are made. This central office or bureau is composed most judiciously of physicians and surgeons who serve in rotation, and who are competent to indicate the particular hospital or ward for the patient, according to the nature of the malady.

The professional charge of the wards in these several hospitals is committed to the ablest men in Paris, and, as already intimated, the appointment is made with reference to the specialty in which the aspirant is believed to excel, or the patients are subsequently so distributed as to accomplish the same object. Two important ends are thus secured; the sick are certain to receive that treatment which will soonest relieve the particular disease under which they may be suffering, the practitioner becoming more and more familiar with his specialty: and those who resort there for instruction, and who will subsequently be called to treat the same malady under less favorable circumstances, find the ablest teachers occupying the chairs in the several departments."

To give some idea of the size of some of the general hospitals, we may state that, Hotel Dieu, La Charity, and the new hospital of Loboriciere, contain each 800 beds, annually affording a home of longer or shorter duration to some 22,000 patients, being so many different specimens of disease. Some of the special hospitals are still larger. Thus the Military Hospital of Val de Grace contains 2,000 beds, whilst Salpatriere appropriated to females affected with diseases considered incurable, or those who are infirm; and to epileptic and lunatic females, furnishes beds for the enormeus number of 4,438. Each hospital has its peculiar attractions. If the student desire to pursue surgery or medicine in a general way, he will very likely attend the clinics of Hotel Dieu or La Charity. The former is rendered immortal by such men as Dupuytren, Bichat, and Laennee, and Boux and Jobert, Rostau and Louis. Here I followed Jobert through a series of operations made according to his *new method* for the cure of that most loathsome of all the accidents which can befall the parturient female, vessico and recto vaginal fistulæ. This eminent man, a great favorite with all the *eleves*, the most brilliant surgeon of the day in Paris, a thoroughly progressive man, was compelled to abandon his post in consequence of mental imbecility supposed to proceed from softening of the brain. Honored by the profession, decorated and made Surgeon-in-Chief to the Emperor, he has fallen a victim to *over work*—the uninterrupted strain upon the nervous system attending the vigorous pursuit of our profession. Although by no means an old man, he recently closed his brilliant and promising career in an asylum for lunatics. Roux, who had performed more capital operations than any living surgeon, was actively engaged in Hotel Dieu on my former visit to this institution. He died some years since, though by special favor he was permitted to continue his connection with the hospital until he was nearly eighty years old. Here also was Louis, the first man to give the world a well digested analysis of any considerable number of cases of carefully noted records of typhoid and typhus fevers.

At La Charity you may still follow Velpeau, who in celebrity is second to no living man, and who attained this great elevation by his own unaided energies, commencing late in life, having been bred a blacksmith. This great surgeon is proud of his origin, and of the fact that he is "self-made." An anecdote illustrative of this point is related of an English snob, who was following Velpeau in his hospital service at a distance, and bestowing but indifferent attention to his instructions, and who remarked to a comrade in a voice loud enough to be overheard by the subject of it, "it's no matter about attending to *him;* he is but the son of a blacksmith." Whereupon Velpeau, piqued at the manner of the cockney's allusion to his origin, retorted, "had you been born a blacksmith you would have remained one still." In October last (1866) I followed this venerable celebrity around his old wards in La Charity. He must now be nearly eighty years old, diagnosing as accurately as ever, applying a bandage as neatly, with his memory perfect, remembering me after an absence of fifteen years, and asking after mutual American acquaintances. Notwithstanding all his learning and long experience, I have seen him make some mistakes, indeed they should be called *blunders*, which would make either of you very uncomfortable if committed in a country town in the "far west," during the first year of your practice. By way of illustration and to show you that everything human may partake of error, I will mention that fifteen years ago I saw him baffled in diagnosing a large aneurismal tumor upon the calf of the leg; and on the same morning saw him run a trochar into, and finally pass a scalpel completely through a scrotum, filled with fungoid growth, in the belief that it was hydrocele; which operation was pronounced by a wag, " a remarkablue specimen of dry tapping." When I wished to see every shade and variety of change in color and thickness, which can be found in the human tegumentary system, I had but to follow Cazzeau or Gebert at St. Louis.

*Hospital du Midi*, the great receptacle for males affected with venereal disease, and for many years presided over by MM. Ricord and Vidal de Casses, ordinarily attracts the attention of all students. It may not be generally known that M. Ricord, at one time lived in this country, and was very poor when he first went to Paris to prosecute his medical studies; although now he is said to be in the receipt of the largest income from his practice of any physician of that city. He is now about sixty-five years old, still vigorous in appearance, of remarkably good judgment, always lectured well, and I never attended his clinic that I did not find the room crowded to suffocation. The Lourcine is designed for the reeeption and treatment of females with the same disease, where I often saw Culerier apply the white hot iron to the uterine neck and orifice, where the affection proved intractable. The venerable Civiale lectured at Hospital Necker, and operated for lithotrity, showing the application of the various apparatus which he had invented for crushing and boring the stone.

Finally, at La Clinique, Maternity, and one of the wards of Hotel Dieu, are to be found at almost any hour, females in the various stages of labor, and where it is not thought offensive to "modesty or common decency" to instruct the young accoucheur in his duties at the bed-side of the patient, or to demonstrate the mechanism of this most important process. Nothing interested me as much on my first visit to Paris as the service and lectures of M. Paul Dubois, at La Clinique; and two or three times each week I followed him with profound attention. To him is conceded the front rank as an obsterician in France, and probably since the death of Niegl̀e, of Heidlebergh, he may be considered the first living operator and practitioner in his department. Notwithstanding his immense private practice, and although entirely independent in his pecuniary condition, he continued his connection with the hospitals, thus adding several hours of severe labor daily to his other arduous duties. In addition to all this, he made frequeut contributions in the way of papers or essays upon subjects connected with his chair, at the weekly meetings of the Academy of Medicine, and still found time to respond to the demands which his large domestic circle and general society made upon him. As the services at the hospitals are rendered for a mere nominal compensation, and could scarcely add to the reputation which he had already acquired, it may be safely concluded that by continuing these voluntary labors he, with many of the names already mentioned, were prompted mainly by a love of science. By keeping alive their zeal for scientific pursuits in the midst of their other avocations as they advance in years, what praiseworthy instances do they afford of industrious perseverence in true scientific investigations, and from the most laudable motives! How worthy of imitation! These are truly men of progress; how unlike many routinists which we behold around us who are pursuing the practice as a mere trade!

Before quitting Paris I want to say that, whilst its schools are among the best in the world in many respects for the accomplished practitioner, they are not entirely safe for the young man who is not well established in orthodox principles. Anatomy may be well learned, but the French are not in my opinion as judicious practitioners as the more intelligent of our own countrymen. Therapeutics are lost sight of in the overweaning anxiety to make a correct diagnosis; and if I can trust my own convictions I would rather a son or young friend of mine should lay the foundation or ground-work of his profession where all the branches are plainly, systematically and thoroughly taught, as in this University, and then have seen a few years' experience before going to Paris, than that he should commence and terminate his studies in the great "ecole de medicine" with all its attractive array of names. Let him first learn to think for himself, to distinguish between fact and theory, to winnow the wheat from the chaff, and thus he is prepared to profit by the mass of material there supplied.

But we will now cross the channel and look in upon the London schools and hospitals for a short time. Here we find the hospitals much smaller, the visits of the several surgeons and physicians much less punctually made, the hospitals without classification into specialties as a general rule, each small hospital becoming the receptacle of every species of disease. Here also clinical lectures are given much less frequently, and almost every hospital has a medical school with a regularly organized faculty attached. Each faculty sells its tickets both for the lectures and the hospitals at rates much higher than in America. Although there are, as you perceive, a multiplicity of medical schools, the pupils must all go before the same board for graduation, and this board is entirely disconnected with the business of teaching. Prominent among the institutions which interested me I might mention King's College Hospital. Here I found Ferguson, who, as an expert operator, is not surpassed by any one in the British dominions.—Here also has been the place of labor of Bowman, the accomplished surgeon and occulist, whose annual income is now estimated at 12,000 or 14,000 pounds sterling. Here, too, is the great Hunterian Museum and its long-time curator, Mr. Queckett, who was perhaps the most expert microscopist in London, and who has written an able work upon the use of the microscope. This museum is the richest in the world in wet preparations and leaves little to be desired. At Bartholomews I often met Mr. Lawrence, Mr. Stanly, and Mr. Paget; and here also I found Mr. West, author of a work upon children, and whom I consider among the most learned men and able teachers, and from whom as much may be expected as from any obstetriciau iu that great city.—Mr. West has been succeeded in St. Bartholomews by Robert Greenhalgh, one of the most active of all the active and progressive men in that department at the present time in London. His wards in St. Bartholomews were always filled with cases of interest to the uterine pathologist, and his instructions at the bed-side are as original and practical as those of any man whom I had the pleasure of meeting during this extensive tour, and his generous hospitality unsurpassed in its princely munificence.

Barnes is at St. Thomas Hospital, whilst Dr. Ramsbotham lectured formerly at St. Georges and Robert Lee had what may more properly be called a lying-in hospital. The intrepid Baker Brown has a private woman's hospital at Notting Hill, where he makes his brilliant operations. The student who wishes to make uterine pathology a specialty will be sure to look up Dr. Henry Bennett, author of a treatise upon "Ulceration of the Neck of the Uterus," etc., etc., in whom they will find a most agreeable and accomplished gentleman. It is well known that in consequence of his introduction of the speculum vagina into more frequent use, and the treatment which he instituted through that instrument with success, he excited the jealousy of the old drones in the profession, and a most bitter persecution on the part of those anti-progress doctors. But, gentlemen, I am happy to say that truth has prevailed in this contest, and Dr. Bennett has long been in the full enjoyment of a very large practice. These virulent attacks have served only to bring him more rapidly and prominently forward, and his opponents have been compelled to resort to this condemned instrument and mode of practice or lose their patients. The older surgeons, Sir B. Brodie and Mr. Lawrence, were still actively engaged in professional pursuits upon my first visit to London. Sir B. declined operative surgery, but was daily consulted by a large number of patients attracted from all parts of the globe by his wide-spread reputation. Mr. Lawrence, notwithstanding his then ample fortune, and immense private practice continued to discharge his duties in St. Bartholomew's Hospital until within the last few months. He had, a few years since, one of the most beautiful places in England, Eeling Park, five miles from the centre of the most fashionable part of London. It contained sixty acres, all of which was in the highest state of cultivation, being laid out into parks and gardens and tastefully ornamented with arbors, walks, arches, fountains and statuary. I was credibly informed that the annual cost of maintaining in their present condition the green-houses and grounds alone exceeded three thousadd pounds sterling, ($15,000.) It should also be added of Mr. Lawrence that he was the embodiment of English hospitality, and was especially attentive to members of the profession who visited London. I have met at his table at the same time medical men from France, Belgium, Hanover and Prussia, which is but a fair illustration of his generous hospitality.

In relation to the practice of the profession in London, it is divided, as you are doubtless aware, into the surgeon, the physician, and general praetitioner. The two first are much the most honorable, but they are attended with great expense in the acquisition and then, without sufficient money to set up a carriage and support a good degree of style, it is exceedingly difficult to obtain business.

Here permit me to ask your attention for one moment to the changes in the relative condition of the profession which have occurred during the last fifteen years in Paris and London. In 1851 the great men of Paris, the men of genius, to many of whom reference has already been made, and a long list of others might be added, were in their glory. Surgery, pathology, uterine pathology, physiology, and every other department was pushed forward by active men, who, each in his sphere, was a *genius* as well as a worker. During this short period these men, Jobert, Roux, Dubois, Cazzeau, Chailly, Morreu, Louis, Hugier, etc., etc., have passed fr om the scene, and for the most part have been succeeded by men of moderate or at most respectable ability, and who without effort to advance the science, are content to post up to the point at which their predecessors left it. It is emphatically the period of *inactivity* in Paris, and as is ever the case, those who are not progressive are lodged like drift wood upon the shore, whilst the current of progress hurries rapidly onward. In London, fifteen years ago, there were a few active men in the department of surgery, physiology and ophthalmology, but in the department in which I felt most interest the profession was non-progressive. Henry Bennett, the pioneer in the doctrine of direct examination and direct application to diseased surfaces, had, it is true, commenced his career in London. He was, however, treated with ridicule by the men in authority, and his views met with few admirers, and still fewer followers. He has lived to see his own complete triumph, and to see those who were loudest in their denunciations compelled to resort to the metroscope or lose their patients. Fearlessly pursuing his investigations he soon placed himself in the front rank in the British metropolis, and had the proud satisfaction within a dozen years to have a long list of able imitators and followers. At this time the speculum is probably used more frequently in London than Paris, and a long list of uterine pathologists have entered the ranks with great energy and success. There is no better illustration of the manner in which London surgeons have distanced the Parisian than is afforded by the comparative frequency of ovariotomy in the former as compared with the latter city. In Paris it is seldom resorted to, and regarded as of doubtful expediency. Whilst Baker Brown, and Spencer Wells and Tyler Smith seldom allow a week to pass without making this operation, which by them, and a iarge number of co-laborers is regarded as *established*, and its propriety no more to be questioned than that for strangulated hernia or hydrocele. More than this, the last operation which I witnessed through the politeness of Spencer Wells, was the seventeenth of a "new series," of which only one had resulted fatally. And Baker Brown had made in my presence the thirty-fourth of a "new series," losing only three of the number. This unparalleled success may not prove to be the rule, but it abundantly demonstrates the propriety, nay the necessity of resorting to it in all cases of ovarian cysts, which sooner or later invariably result in death. Those who have done me the honor to listen to my lectures will recollect that, for many years I have advocated the feasibility of this operation, and pronounced it as proper as amputation of the thigh; and advanced statistics tc show that its fatality was less than in those amputations, the propriety of which no one pretended to question.

Not many days before leaving Paris a friend called upon me for advice in relation to a case of ovarian tumor, which by Velpeau, Nelaton and other eminent French surgeons had been pronounced irremediably fatal. I did not for one moment hesitate to differ with these distinguished men, insisting they were behind the intelligence in this matter, advised the sending for Marion Sims, and by no means to abandon the case as hopeless. An operation was immediately decided upon, which was to be made by Dr. Sims the Wedesday after my departure for America. Now this individual operation may not succeed, but in London or New York it would be deemed inexcusable supineness to allow the poor woman to die without an effort for her rescue. The same, or much the same remarks, would hold true in all the departments in which recent advance has been made. Brown, and Wells, and Barnes, and Tyler Smith, and Greenhalgh, and West, and Meadows, and Oldham, with Bennett and Tilt, make an amount of talent rarely devoted to the advancement of one department at the same time, and they are advancing with giant strides, whilst Paris remains nearly stationary.

But we must pass on, and will ask your attention for a short time to the University of Dublin. It is very richly endowed, having a beautiful arrangement of buildings, immense library and faculties of great ability in all its departments. Here, too, is the best lying-in hospital in the British dominions. Here such men as Churchill, and Collins, and Johnson, and Shackleford, and Mc Clintock, demonstrate the process of parturition upon the living subject in the belief that the cause of science and humanity are alike promoted by it. The large, general hospital, St. Steephens, has an array of talent in its staff seldom exceeded. I have seen Sir Philip Crampton, Mr. Cusic and Mr. Colles, aiding each other in the removal of the upper jaw, cutting for stone, and in other operations; whilst in the medical department Stokes, Graves and Flemming, were all concerting for the welfare of the same patient. I may add that in intelligenee, in high-minded, generous, fraternal coöperation, in the zeal with which scientific investigations are pursued, in the absence of that opprobrium of the profession, jealousy; and in liberal hospitality, the Medical men of Dublin are not surpassed by any similar body of men in any country. The younger members of the profession are, however, very poor in Ireland; and it often requires a very long, hard struggle, to obtain business sufficient to afford a subsistence.

Passing by Glasgow, as containing nothing worthy of note in this brief sketch, let us proceed at once to the ancient and renowned Scotch metropolis, Edinburgh. This venerable city was more celebrated for its medical school fifty years ago than any in the British dominions, and scarcely second to any in the world. This institution is at this time in a flourishing condition, with an able faculty, and well deserves the attention of the medical traveler. Mr. Syme, Sir Geo. Ballingal and Mr. Millar, have each a world-wide reputation as surgeons, and Dr. Bennett is a most indefatigable and successful teacher of microscopic anatomy. Here also is to be found Dr. Christison, scarcely inferior to the great French authority, Orfila, upon poisons, or in anything indeed pertaining to chemistry. But in my own department will be found Prof. Simpson, (now Sir James,) ready to describe more new theories and show more novel instruments for the treatment of affections of the female genital organs than any one whom it was ever my good fortune to meet. He adds to great originality and genius, a ready and discrim inating judgment. He is scarcely of medium height, strongly built, with a physical development capable of enduring great fatigue, and is able to accomplish more in the twenty-four hours than any man of whose habits I have any knowledge. He has an immense private practice, performs daily his service at the hospital, delivers his lecture at the college with great punctuality, is a regular attendant at the meetings of the societies, and seldom without a paper of interest to read; publishes more original articles or monographs than any other obstetrician, and yet finds time for carrying forward physiological investigations, making experiments upon inferior animals, and still more strange amidst these multifarious occupations, has time to write dissertations upon subjects connected with ancient literature. Again, he has been collecting facts and writing a work upon ancient medical seals, a subject entirely foreign to his medical pursuits, and which in point of merit, it is said would do credit to a professed antiquarian. Notwithstanding all this, if I may judge by my own experience, I would pronounce him the most attentive to strangers in the same department of medicine. and the most hospitable man in Europe.

Leaving Edinburgh we will cross over to Holland. Visiting the old Universities of Haarlem and Utrecht I found but the shadow of their former greatness left. Richly endowed, with ample apparatus, extensive museums and the prestige of long established reputation; situated in small towns, without the means of clinical instruction; they are dwindling into insignificance in consequence of the superior hospital privileges afforded at Amsterdam. At the latter place also there are two lying-in hospitals, and in Holland as in most countries of Europe, at the present day, attendance at these institutions is required before they will admit the student to an examination for his degree.

Proceeding up the Rhine, visiting the large and well regulated hospital at Cologne, I next halted at the University of Bohn. This modern school is an especial favorite with the Prussian government, being liberally endowed by it, and is deservedly popular. Saw Prof. Kilian upon my first visit to this University, with whom I visited his maternity and the extensive gardens attached, and his extensive museum of obstetric curiosities. I should not omit to mention that Prof. Kilian has made the cesarean section twelve times, and in just one-half he has saved both patients. He was kind enough to send to my hotel for examination the wife of a shoe-maker, with deformed pelvis, who, in addition to the pelvic deformity, showed the eschars of four divisions of the abdominal parieties. Two of the children thus extracted survived, and she was now pregnant for the fifth time.

From Bohn, after viewing the beautiful scenery of the Rhine, I directed my course to the University of Giessen, rendered celebrated in modern times by Liebeg, who was actively engaged in teaching. He is a middle aged, unpretending man, and would seem to owe his success rather to persevering industry than to his genius. Liebeg has since been transferred—is no longer to be found at Giessen. Called upon Prof. Retgen, who showed me his extensive cabinet of obstetric instruments, his maternity, and what was of much more interest, his collection of deformed pelves, which are said to be the most extensive in existence. He has every description of deformity, several of the oblique deformity, known as that of Neiglè, and which is very rare. Prof. R. is of opinion that this deformity often supervenes in after life. He has one specimen which is anchilosed at the sacro-illiac junction upon the side deformed, and henee he argues that it is impossible that it could have boen congenital.

Berlin, with its university and hospitals, is the next point of interest which claims attention. The lying-in establishment is under the celebrated accoucheur, Prof. Busch. The building is sufficiently extensive to accommodate more than one thousand women each year. They are all delivered upon a frame in the form of a bed when extended, in which the centre of the foot can be removed. It is covered with leather, stuffed with curled hair, and there are places on either side for the support of the feet of the female who is placed upon her back. The female, I need scarcely add, is so placed that all the students may witness the attending phenomena and the manner in which the accoucher discharges his duties. Every Tuesday and Friday the pupils are practiced in the toucher and in auscultating the intro-uterine sounds. Langenbeck has succeeded Dieffenbach as clinical teacher of surgery, and is perhaps at this time scarcely inferior in learning or in any of the qualities necessary to make a great surgeon, to his illustrious predecessor. Nothing which he was polite enough to show interested me more than the cases of resection of bones, of which there were several there present, and all of which seemed doing well.

During my stay in Berlin I often met a short, heavy looking man, with his eyes so prominent as to seem almost a deformity, whom you would set down for the dullest man in the city. This was Muhler, the great physiologist. The moment he commences conversation his countenance lights up with intelligence undergoing a complete change. Passsed much time with Dr. Caryl Meyer, who may be styled the Simpson of Berlin. At his private hospital I saw many curious instruments of his own invention for the treatment of prolapsus, retroversion, etc., some of which will be shown you during the ensuing course. Found the accoucheurs of Berlin still using the clumsy obstetric forceps of Siebold, which have been discarded in France and this country long since. The museum of the University of Berlin is very rich in rare specimens in human, comparative and morbid anatomy. Among other objects my attention was called to the skulls of two brothers, who lived to adult age, with tolerable physical development, which were scarcely larger than the fœtal or infantile size, although firmly ossified. I was informed that when living, they were exceedingly stupid, being but little above the brute creation in intelligence. There are in the Berlin museum also many interesting specimens of headless monsters, hermaphrodites with remarkable developments, and several specimens of double uteri and vaginæ. Upon the whole, judging from what I saw of the medical men and schools of Berlin I am impressed with the belief that they will scarcely suffer by comparison with those of any other city of Europe.

On the way to Vienna, Prague may be visited, which contains the oldest Uiniversity in Germany. It was founded by the Emperor Charles the IV, in 1348. The fame of the teachers of this

University, and the privileges granted to scholars soon attracted hither students from all parts of Europe, until the number is said not to have been less than 40,000. The religious difficulties, known as the "Hussite rebellion," in connection with an abridgment of the privileges of the foreign students drove from it in one week the enormous number of 25,000. These men dispersed themselves over Europe, and became the founders of the Universities of Liepzig, Hiedleberg and Crawcow. The literary department of this ancient school still maintains a high reputation, with one of the richest libraries on the continent. The medical department, though respectable, is yearly losing ground in consequence of the superior privileges afforded in Vienna for clinical instruction. Kivish died before my visit to Prague.

Continuing the narrative, we come next to the Austrian metropolis. In Vienna the medical student will find the medical faculty thoroughly educated, a University well organized, with all its appointments judiciously made, with one of the richest museums in the world, and the most extensive hospital which I have ever visited. Some idea of the industry of the German student may be gained by knowing that in addition to his knowledge of the modern languages he is able to speak and write the Latin with facility. Indeed, until recently, the lectures have been delivered and examinations for degrees carried on entirely in Latin. It is also certain that in every department of medicine they are thoroughly posted and actively engaged in new investigations. In pathology they are second only to the Parisians, and in chemistry, animal and vegetable, the Germans have placed themselves in advance of all the European schools by their recent researches and discoveries. In clinical teaching the hospitals of Vienna afford a most extensive field which is fully improved. Much more attention is paid to the *treatment* of the various diseases submitted to the observation of the pupil, though it can scarcely be possible to exceed the French in description of symptoms and accuracy of diagnosis. The General Hospital is an immense establishment, having no less than 111 wards or *salles*, and capable of accommodating at one time more than 4,000 patients. These several wards are arranged around many squares or parks, in the centre of each of which there is a fountain constantly playing, having beautiful walks also with ample shade for the convalescents. Each court has its specialty, in one is to be found Prof. Rosas, with abundant subjects for illustrating the state of ophthalmic science in Vienna. In another Prof. Sigmund presides over the wards for the reception of syphilitic diseases, in the treatment of which he is second only in reputation to M. Ricord. In another (all being entirely separated by means of heavy gates) are the wards of Dr. Chiari, which possess peculiar interest for the obstetric practitioner. Into these rooms are received all females who are ill with any post partum disease. There is a separate room for those who may be attacked with puerperal fever, which is not connected with any other rooms, and the utmost precautions are taken to prevent its extension by contagion. In the wards of Dr. Chiari are to be found every conceivable affection which can afflict the woman after child birth. Most of all will the young practitioner be attracted by the immense number of labors in the lying-in wards under the care of Profs. Klein and Beraun. Here are to be found ordinarily several women in labor in the salle de accouchments at any hour of the day. There being seldom less than fifteen or twenty, and sometimes as many as forty labors in the twenty-four hours. The number of females annually accouched here amounts to 6,000 or 8,000. The institution is in good repute among the people, it being considered a favor by those in comfortable circumstances to gain admission. Here the student may witness all the different kinds of labor, natural and artificial, which he will be likely to encounter in after life. Here, then, as in all of the Universities of Europe, the student is taught his duties to the partuerient as well as to his surgical and general patient, *at the bed-side*, it not being there thought less important that he should understand these duties when two lives are jeopardized than one, and that one often at best left mutilated by the operation. Nor is it expected that he will be gifted with superhuman knowledge for practice in this department only. Indeed I am daily more and more convinced of the importance of clinical instruction in obstetrics, which is now nowhere properly afforded in this country, and cannot but believe that the day is not distant when truth and the cause of humanity and sound education will dispel the clouds which have been raised by a sinister and prejudiced appeal to the passions of the multitude.

In Vienna is to be found the most extensive collection of preparations in wax to be met with in Europe, except that of the Grand Duke of Tuscany at Florence. And here I may for the benefit of those who may wish to purchase illustrations, remark; that after careful investigation in Germany and Italy, I am convinced that they are better made, at less cost, in Paris, than in any other part of Europe.

But, gentlemen, time will not allow me to follow farther the route pursued, and I must draw these remarks to a close, simply assuring you that it shall be my effort to lay before you, in due time, during our future intercourse, whatever observations I have been able to make bearing upon the department which it is my duty to bring to your consideration.

